# Microbiological Surveillance of Biogas Plants: Targeting Acetogenic Community

**DOI:** 10.3389/fmicb.2021.700256

**Published:** 2021-08-16

**Authors:** Abhijeet Singh, Jan Moestedt, Andreas Berg, Anna Schnürer

**Affiliations:** ^1^Anaerobic Microbiology and Biotechnology Group, Department of Molecular Sciences, Swedish University of Agricultural Sciences, Uppsala, Sweden; ^2^Tekniska Verken i Linköping AB, Department R&D, Linköping, Sweden; ^3^Gasum, Linköping, Sweden

**Keywords:** anaerobic digestion, acetogens, formyltetrahydrofolate synthetase, high-throughput sequencing, community profile

## Abstract

Acetogens play a very important role in anaerobic digestion and are essential in ensuring process stability. Despite this, targeted studies of the acetogenic community in biogas processes remain limited. Some efforts have been made to identify and understand this community, but the lack of a reliable molecular analysis strategy makes the detection of acetogenic bacteria tedious. Recent studies suggest that screening of bacterial genetic material for formyltetrahydrofolate synthetase (FTHFS), a key marker enzyme in the Wood-Ljungdahl pathway, can give a strong indication of the presence of putative acetogens in biogas environments. In this study, we applied an acetogen-targeted analyses strategy developed previously by our research group for microbiological surveillance of commercial biogas plants. The surveillance comprised high-throughput sequencing of FTHFS gene amplicons and unsupervised data analysis with the AcetoScan pipeline. The results showed differences in the acetogenic community structure related to feed substrate and operating parameters. They also indicated that our surveillance method can be helpful in the detection of community changes before observed changes in physico-chemical profiles, and that frequent high-throughput surveillance can assist in management towards stable process operation, thus improving the economic viability of biogas plants. To our knowledge, this is the first study to apply a high-throughput microbiological surveillance approach to visualise the potential acetogenic population in commercial biogas digesters.

## Introduction

Biogas generation is a very versatile process, in which almost any biodegradable material can be used to produce biogas and biofertiliser (Schnürer, 2016; Schnürer and Jarvis, 2017). It involves a complex and interdependent anaerobic microbiological consortium working in synergy to carry out hydrolysis, acidogenesis, anaerobic/syntrophic oxidation and methanogenesis (Schnürer, 2016; Borja and Rincón, 2017; Robles et al., 2018; Kleinsteuber, 2019; Theuerl et al., 2019). Thousands of known and unknown microbial species cooperate and coordinate in the biogas process, making it very different from other industrial fermentation processes (Wolf et al., 2009; Madsen et al., 2011; Drosg, 2013; Ferguson et al., 2014; Maus et al., 2016; Treu et al., 2016; Campanaro et al., 2020; Yoshida and Shimizu, 2020). This microbiological complexity lowers the scope for automatic control and optimisation, making the process prone to unintended changes in performance and stability (Ward et al., 2008; Wolf et al., 2009; Madsen et al., 2011). For adequate use of the resources invested in commercial biogas production, process optimisation and constant monitoring of the process are extremely important (Madsen et al., 2011; Drosg, 2013; Schnürer, 2016). Various physical and chemical analysis technologies are available for monitoring the biogas process, but they are based on consequential parameters and are not completely reliable in predicting disturbances in microbial communities (Ward et al., 2008; Ferguson et al., 2018; Yoshida and Shimizu, 2020). Therefore, new methods are needed for constant monitoring of microbiological community structure and dynamics in biogas reactors (Fernández et al., 1999; Drosg, 2013; Ferguson et al., 2014).

The whole microbial aggregation is important in the synergistic coordination required for the biogas process (Schnürer, 2016; Kleinsteuber, 2019). Among the microbial communities involved, the acetogenic community is critical in synchronising and balancing the biogas process and acts as a connecting link between hydrolysing/fermenting bacteria and methanogens (Kovács et al., 2004). Despite their importance and versatility in biogas generation, the acetogens remain neglected in microbiome-oriented studies on the anaerobic digestion process (Theuerl et al., 2019). Studies using advanced technologies, such as metagenomics, metatranscriptomics, metaproteomics, etc., have demonstrated that biogas microbiomes are highly diverse and that each biogas reactor develops its specific microbial community based on the substrate/s and operating parameters (Schlüter et al., 2008; Hanreich et al., 2012; Heyer et al., 2013, 2016; Kohrs et al., 2014; Campanaro et al., 2016; Güllert et al., 2016; Luo et al., 2016; Maus et al., 2016; Ortseifen et al., 2016; Treu et al., 2016). Large-scale omics studies have been paramount in unravelling microbial dark matter, but none to date has focused on the structure or diversity of specifically acetogenic communities or examined the importance or functional role of this vital group in biogas reactors.

Some targeted amplicon-based studies have examined the acetogenic communities in biogas reactors and other environments (Leaphart and Lovell, 2001; Pester and Brune, 2006; Ohashi et al., 2007; Gagen et al., 2010, 2014; Henderson et al., 2010; Akuzawa et al., 2011; Hori et al., 2011; Westerholm et al., 2011a, 2018; Moestedt et al., 2016; Müller et al., 2016; Li et al., 2017; Saheb-Alam et al., 2017). These studies employed the formyltetrahydrofolate synthetase (FTHFS) gene, which is a marker for acetogenic bacteria but were performed using techniques, such as clone library or T-RFLP profiling, yielding limited information about acetogenic community structure and diversity, taxonomic identities, temporal changes, etc. This was due to the lack of tools and methods that could be used efficiently and reliably to gain a deeper understanding of acetogenic communities and their characteristics. Recent developments, such as the creation of the acetogen-specific database AcetoBase (Singh et al., 2019) and the high-throughput data analysis pipeline AcetoScan (Singh et al., 2020), have helped significantly in facilitating the in-depth analysis of potential acetogenic communities. Moreover, a recent comparative study demonstrated the superiority of a FTHFS gene-based sequencing method over FTHFS gene-based T-RFLP or 16S rRNA gene sequencing in targeting acetogenic community structure and community dynamics (Singh et al., 2021a).

The aim of the present study was to use a FTHFS gene-based high-throughput sequencing method for microbiological surveillance of potential acetogenic communities in commercial biogas plants in Sweden. For this, long time series of weekly samples from full-scale biogas reactors operating with different feed substrates (food waste, sludge, manure, green waste, etc.) and operating conditions were used. To our knowledge, no previous study has successfully devised and applied an acetogenic community-oriented microbiological surveillance strategy for biogas plants.

## Materials and Methods

### Sample Collection and Processing

Samples were collected weekly for several years from six Swedish biogas plants with different operating conditions [continuous stirred-tank reactor (CSTR) or plug flow, organic loading rate (OLR), hydraulic retention time (HRT), temperature, etc.] and different substrates (e.g., food waste, food waste with sludge, agricultural waste, manure, sludge, etc.; Table 1). Volatile fatty acid (VFA) analysis, including C2–C6 acids, was performed by the respective biogas plant operator and process metadata (OLR, HRT, temperature, gas yield, methane content, ammonium-nitrogen, pH, VFA, etc.) were provided. The VFA and ammonium-nitrogen analyses were mainly performed using methods described by Moestedt et al. (2016). The period with no VFA accumulation was defined as the stable phase, the period with VFA accumulation as the disturbance phase, and the subsequent phase, where the accumulated VFA were degraded, as the recovery phase. Disturbance was characterised by varying levels of volatile acid accumulation (2–17 g/L). Samples for microbiological surveillance were selected using a disturbance-centred strategy, with samples collected from the stable phase preceding disturbance, during the disturbance phase and in the recovery phase. For DNA extraction and library preparation, single replicates from the stable phase and two or three replicates from the disturbed phase over an extended time series were selected. The genomic DNA and sequencing library were prepared as previously described (Singh, 2020; Singh et al., 2021a). All samples were pooled, with an equal amount from each (20 ng), and paired-end sequencing was carried out on Illumina MiSeq with v3 chemistry at the sequencing facility of the SNP&SEQ technology platform in Uppsala (UGC, 2018). For multiplexed high-throughput sequencing, a total of 391 samples were used in two separate sequencing runs, from which the data were combined for analysis.

**Table 1:** Characteristics of the six Swedish commercial and industrial-scale biogas reactors sampled for microbiological surveillance in this study.

No.	Reactor code	Reactor type	Substrate	Avg. biogas (Nm^3^ d^−1^)	Total VFA (g L^−1^)	pH	N-NH_4_^+^ (g L^−1^)	Temp. (°C)	OLR (gVS L^−1^ d^−1^)	HRT (Days)
1^*^	C1-DD1	Plug-flow	Food waste	1,683	0.4–17	7.4–8.0	4.0–6.6	38–40	5.7–9.3	30–49
2^†^	C2-VX1/2	CSTR	Food waste + sewage sludge	2,102	0.0–3.2	7.5–7.9	1.2–2.3	39–41	3.2–3.4	18–29
3^†^	C3-K1/2	CSTR	Cow/pig manure, cereals, silage and fats	7,375	0.0–0.3	7.5–8.0	2.6–4.2	36–38	1.1–4.9	30–68
4^†^	C4-VS1/2	CSTR	Horse/chicken/pig manure and cereal husks	4,543	0.0–1.2	7.6–7.9	2.6–7.9	36–38	0.8–3.8	23–103
5^†^	C5-A1/3	CSTR	Food/slaughterhouse waste	11,937	0.0–0.2	7.5–8.2	2.2–3.4	38–42	0.2–3.9	27–50
6^†^	C6-O1/2	CSTR	Stillage, grass silage and plant residues	10,255	0–3.3	7.3–7.9	2.9–3.7	36–40	3.4–7.7	45–111

*Values included in the table are metadata provided by respective biogas plant operator*.

* *Plug-flow dry digestion type biogas reactor*.

† *Parallel biogas reactor. Average values/range of each parameter are provided here, for exact values see supplementary material*.

### Sequence Data Analysis

Unsupervised FTHFS gene sequence data analysis was performed using the AcetoScan pipeline (1.0; Singh et al., 2020). Before the analysis, a single file was created by concatenating the raw forward and reverse read files for each sample. The parameters used for the AcetoScan analysis were −m 300, −n 150, −q 20, −c 5 and −e 1e–30, while for other parameters default settings were used (AcetoScan users’ manual). Customised visualisation of the AcetoScan results was done using the packages *phyloseq* (1.30.0; McMurdie and Holmes, 2013) and *vegan* (2.5.7; Oksanen et al., 2019) in R (3.6.3; R Core Team, 2013) and RStudio (1.3.1073; RStudio Team, 2020). All data processing and visual analyses were performed on a Debian Linux-based system with x86_64 architecture and 3.4 Ghz Intel® Core™ i7-6700 processor.

## Results

### Reactor Characteristics

The biogas reactors that supplied samples for the analysis differed in their operating strategies and feed substrates (see Table 1). Except for reactor C1-DD1, all reactors were parallel reactors at the specific plant (C2–C5). During the sampling period, some fluctuations in feed ratio, temperature and OLR and in associated consequential parameters, such as methane and carbon dioxide content (%), pH, etc., were observed (Table 1). Reactor C1-DD1, a food waste plug flow reactor, reached a very high level of VFA accumulation (~17 g/L), but only minor VFA accumulation (2–3 g/L) was observed for the CSTR reactors C2-VX1/2, C3-K1/2, C4-VS1/2 and C6-O1/2, and no accumulation of VFA was observed in C5-A1/3.

### High-Throughput Sequencing

The size (compressed) of combined forward and reverse reads from two separate runs was ~6.5 and ~7.1 Gb, respectively. Since the forward and reverse reads cannot be paired, the forward and reverse read fastq files for individual samples were merged in a single file. The overall final size of the merged fastq data files was ~14 Gb and ~61 M sequences. After quality filtering ~35 M sequences were used for clustering at 100% identity, which resulted in 1897 OTUs.

### Potential Acetogenic Community Structure

**Figure 1:**
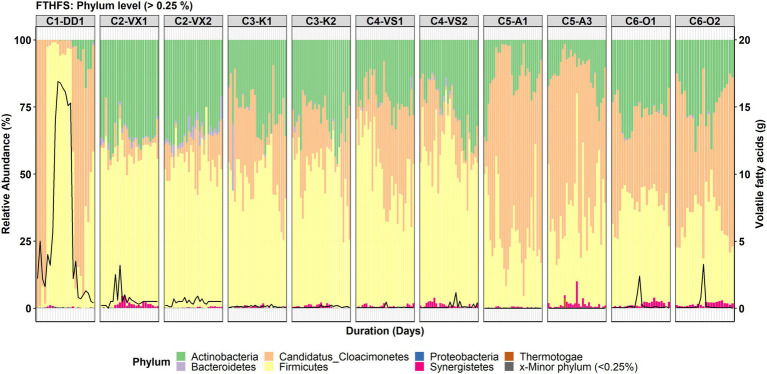
Phylum-level community structure in the six reactors (C1–C6) visualised with formyltetrahydrofolate synthetase (FTHFS) amplicon sequencing. The black line represents the concentration (g/L) of total volatile fatty acids.

The total number of taxa detected in community-level analysis for the classification levels from phylum to species was 11, 23, 32, 52, 106 and 152, respectively. It should be noted that for C1-DD1, the initial samples (1–4) were taken from a stable period longer than 1 year (with low VFA levels), whereas the following samplings were in the VFA accumulation phase (Figure 1). However, community structure was similar in the phases before and after VFA accumulation.

#### Phylum-Level Community Structure

The top three most abundant phyla detected in all the reactors were Firmicutes, Candidatus Clocimonetes and Actinobacteria (Figure 1). At phylum level, the changes in community structure during VFA accumulation were most distinct for C1-DD1. Candidatus Clocimonetes was the most abundant phylum in the stable and recovery phases, but during the period with VFA increase the community structure drastically changed and Firmicutes was instead the dominant phylum, representing >90% of total relative abundance (RA; Figure 1). The phylum-level community structure for C2-VX1/2 showed the highest abundance of Firmicutes (RA ~50–60%) and Actinobacteria (RA ~25–35%), followed by Candidatus Clocimonetes (RA ~1–20%), Bacteroidetes and Synergistetes. The phylum-level community structure in C3-K1/2 and C4-VS1/2 was found to be similar with respect to the top three phyla, *viz*. Firmicutes (RA ~40–50%), Actinobacteria (RA ~20–40%) and Candidatus Clocimonetes (RA ~5–20%). High abundance of Candidatus Clocimonetes (RA ~10–90%) compared with Firmicutes (RA ~8–40%) and Actinobacteria (RA ~2–35%) was also observed in C5-A1/3. In reactor C6-O1/2, the top three phyla were Firmicutes, Candidatus Clocimonetes and Actinobacteria, which showed similar RA.

#### Genus- and Species-Level Community Structure

**Figure 2:**
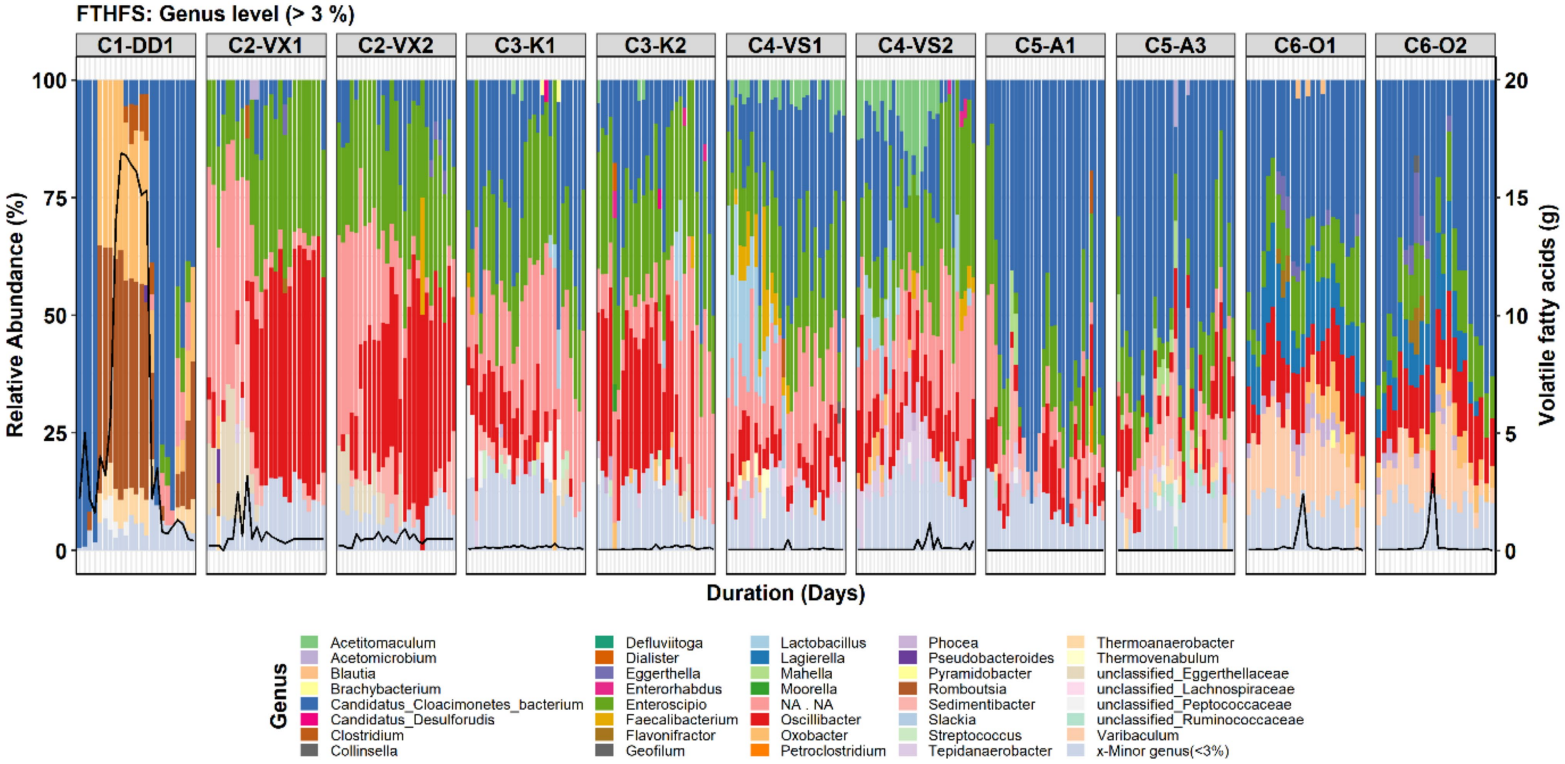
Genus-level community structure in the six reactors (C1–C6) visualised with FTHFS amplicon sequencing. The black line represents the concentration (g/L) of total volatile fatty acids.

A total of 106 genera were observed in the genus-level community analysis, but in low abundance, with only 39 and 32 genera having RA >3 and >5%, respectively. At species level, 152 species we observed, of which 48 and 37 species had RA >3 and >5%, respectively. Overall, Candidatus Cloacimonetes bacterium was the most abundant genus detected (Figure 2). It was also the most abundant genus in C1-DD1, before and after VFA accumulation, and in C5-A1/3 and C6-O1/2. However, in reactor C2-VX1/2, *Enteroscipio* and *Oscillibacter* were instead the most abundant genera, except in the first period of surveillance, where this reactor was enriched with uncultured Firmicutes bacterium. Other genera identified at relatively high abundance (RA >3%) were *Lagierella* and *Varibaculum* in C6-O1/2 and *Tepidanaerobacter*, most significantly (RA >3%) in C4-VS1/2 (Figure 2).

**Figure 3:**
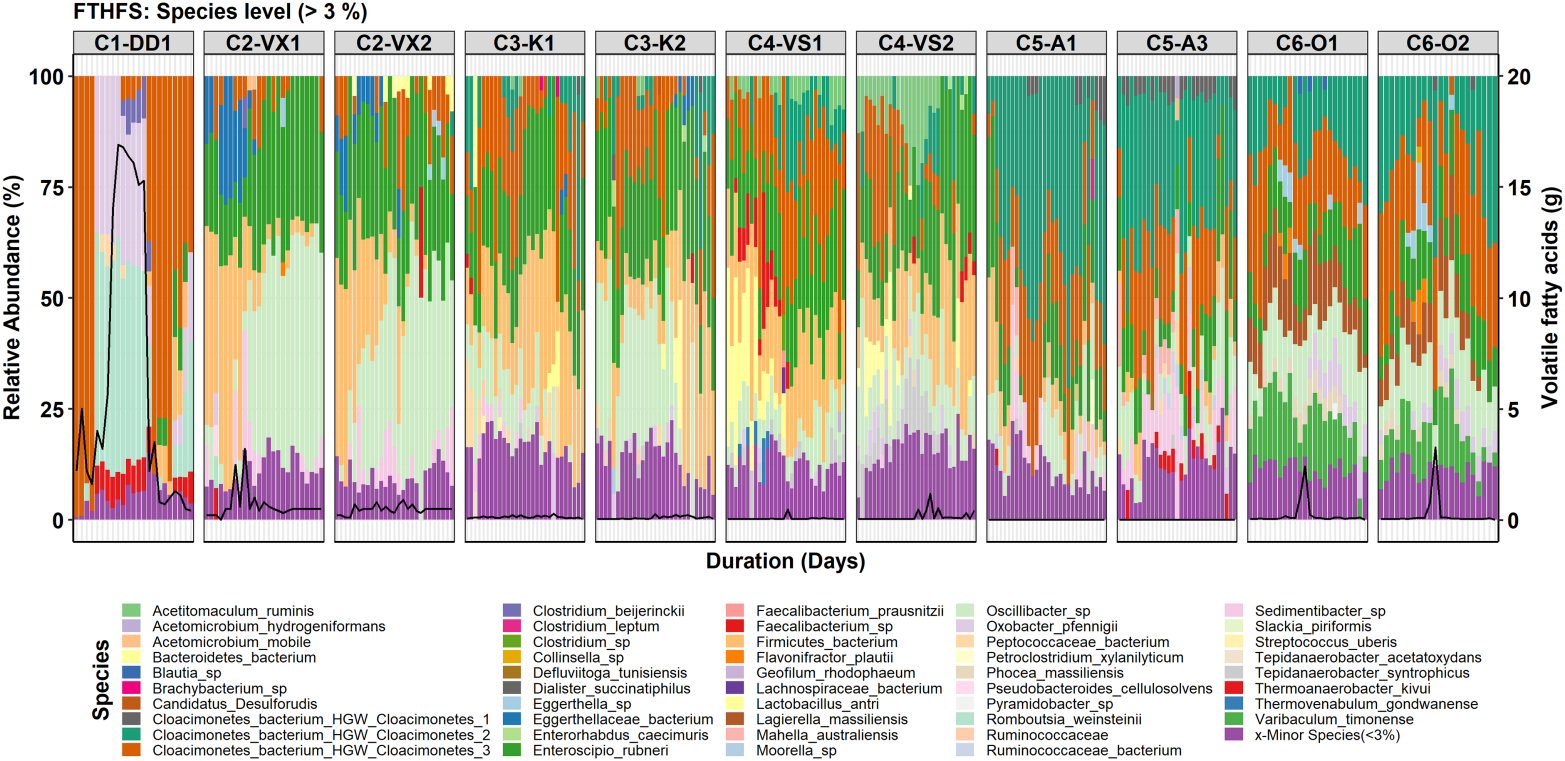
Species-level community structure in the six reactors (C1–C6) visualised with FTHFS amplicon sequencing. The black line represents the concentration (g/L) of total volatile fatty acids.

For the species-level community analysis, the threshold used for visualisation was 3% RA. The results showed that the Candidatus Cloacimonetes bacterium in the different reactors was represented by three different species (Figure 3). In reactor C1-DD1, Cloacimonetes bacterium HGW_Cloacimonetes_3 (Cloacimonetes_HGW3) was the only species detected within the phylum Candidatus Clocimonetes. With the increase in VFA levels in reactor C1-DD1, the RA of this genus fell below the 3% threshold, in parallel with appearance and increasing RA of the species *Romboutsia weinsteinii* and *Oxobacter pfennigii*. In C5-A1/3, the species Cloacimonetes_HGW1 was instead detected (RA >3%). The species Cloacimonetes_HGW2 was detected (RA >3%) in all reactors except for C1-DD1 and C2-VX1/2. Other species detected were *Mahella australiensis*, observed in C5-A1/3, *Lactobacillus antri*, detected in C3-K1/2 and C4-VS1/2, and *Thermoanaerobacter kivui*, detected in reactors C1-DD1 and C5-A1/3. *Clostridium beijerinckii* was most significantly observed during the high VFA levels in reactor C1-DD1 (Figure 3).

**Figure 4:**
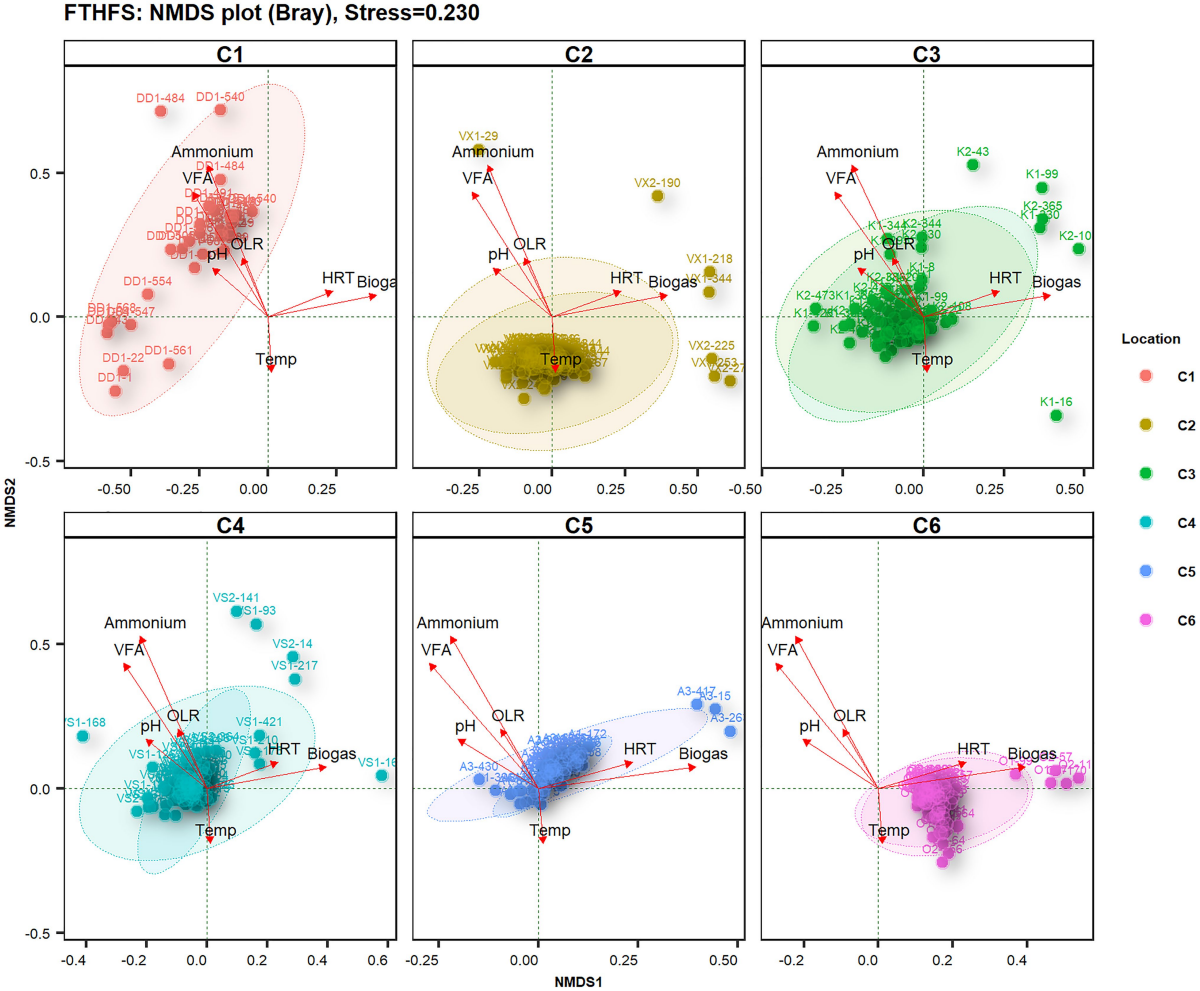
Beta-diversity of samples from the six reactors (where C2–C6 are parallel reactors) operated with different substrates visualised with FTHFS amplicon sequencing and non-metric multidimensional scaling (NMDS) analysis with Bray distances.

Non-metric multidimensional scaling (NMDS) diversity analysis with Bray distances helped to visualise differences in microbial diversity in samples from the different biogas reactors under the influence of different operating parameters (Figure 4). The most influential driving parameters for microbial community structure in reactor C1-DD1 were ammonium and VFA level. The temperature was the most influential environment variable for microbial community structure in reactor C2-VX1/2. Reactors C3-K1/2 and C4-VS/2 did not show any specific significant sample dispersal under any environment variable. The samples from reactor C5-A1/3 were observed to be influenced by OLR and HRT, while those from reactor C6-O1/2 were influenced by the combined effect of HRT and temperature.

**Figure 5:**
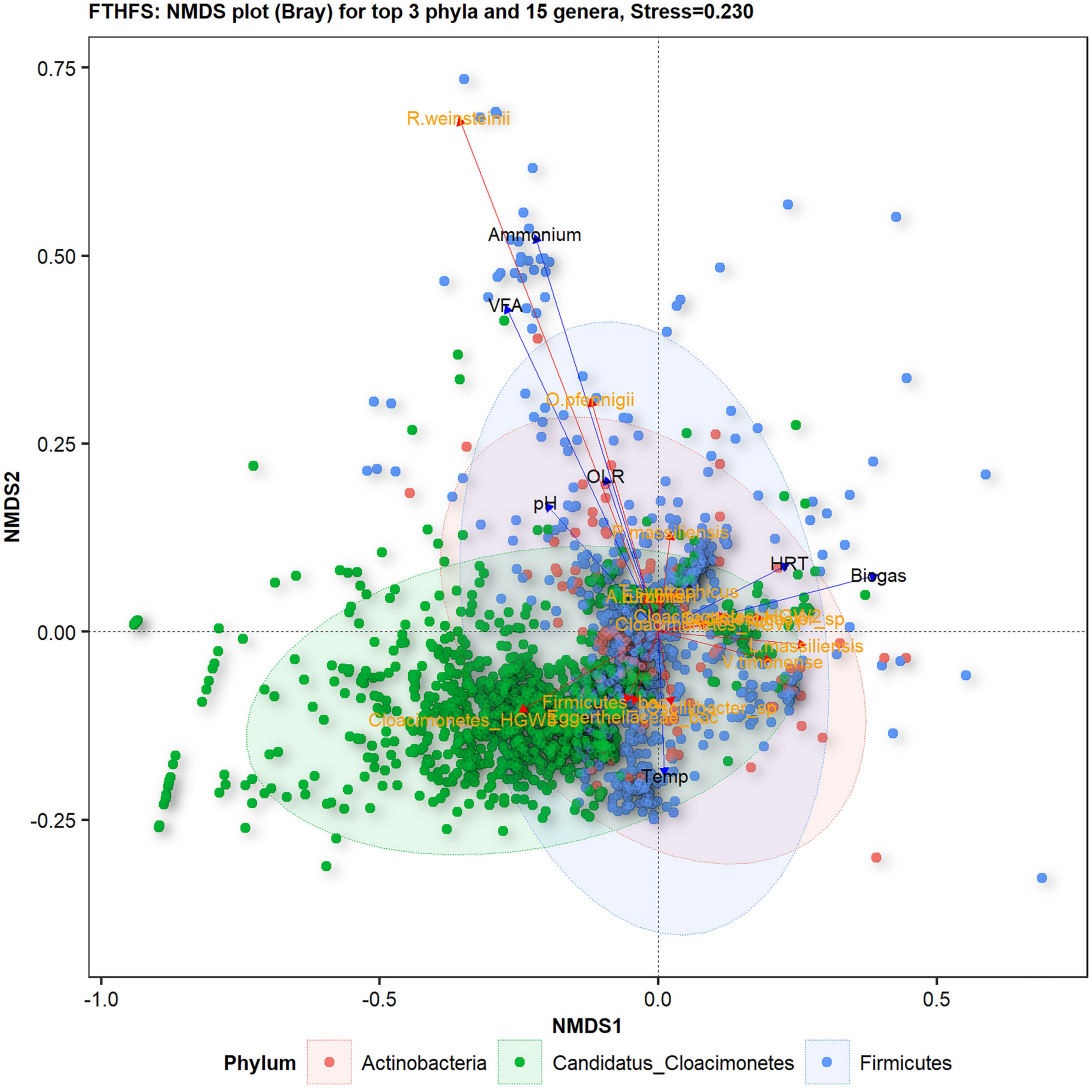
Beta-diversity of the top 15 genera belonging to the top three phyla from in reactors (C1–C6) operated with different substrates, visualised with FTHFS amplicon sequencing and NMDS analysis with Bray distances.

Based on NMDS analysis with Bray distances on samples from the different reactors, the top 15 species from the top three phyla showed relationships with physico-chemical changes (Figure 5). The species *Romboutsia weinsteinii* and *Oxobacter pfennigii* were found to be positively related to ammonium and VFA levels in the reactor, whereas Cloacimonetes_HGW3 appeared to be most sensitive to changes in physico-chemical parameters, particularly OLR, VFA and ammonium. *Lagierella massiliensis* and *Varibaculum timonense* were positively influenced by temperature and HRT. Uncultured Eggerthellaceae bacterium and Firmicutes bacterium showed negative relationships with VFA levels.

## Discussion

Community profiling analyses using FTHFS gene amplicon sequencing revealed potential acetogenic community structure, temporal dynamics and the influence of environmental variables on the microbial diversity in biogas reactors. Different reactors were operated with different feed substrates and operating conditions (Table 1), which resulted in differences and some similarities in overall community profile and dynamics. However, the similarities and difference in community structure could not be directly associated to the substrate type. The reactors used in this study were operated with mixed waste, which might explain why a clear substrate-specific clustering of samples or taxa were not observed (Figures 4, 5). The main driver of the community structure appeared instead to be the ammonium levels in different reactors. This result is in concordance with the previous studies of the biogas microbiome targeting 16S rRNA, suggesting that ammonia is a strong driver for clustering the community (De Vrieze et al., 2015). The most distinct features of the microbial communities in the individual reactors, in association with the environmental parameters, are further discussed below.

Reactor C1-DD1, a high solid plug flow system operating at high loads using food waste as substrate showed drastic changes in community structure during the sampling period. This could have been partly due to the large sampling gap (385 days) between samples 4 and 5, but was more likely caused by an increase in VFA level around sampling point 7, caused by an increase in ammonium-nitrogen level (to above 6 g/L). Ammonium-nitrogen concentrations of this level have previously been shown to cause a significant inhibition of the biogas process and VFA consumption (Moestedt et al., 2016; Schnürer and Jarvis, 2017). Moreover a shift in the potential acetogenic community in response to increasing ammonia level and a shift to syntrophic acetate oxidation have been observed before targeting FTHFS gene in TRFLP analyses (Müller et al., 2016). The species *Romboutsia weinsteinii*, *Thermoanaerobacter kivui* and *Oxobacter pfennigii* were the most prominent species in C1-DD1 when the VFA levels were high, and with the appearance of *Clostridium beijerinckii*, a drop in VFA levels was seen. It should be noted that *Thermoanaerobacter kivui* and *Clostridium beijerinckii* are known acetogens (Drake et al., 2008; Singh et al., 2019), while *Romboutsia weinsteinii* and *Oxobacter pfennigii* are suggested to be acetogens (Collins et al., 1994; Nierychlo et al., 2020). *Romboutsia* was previously observed to be a misclassification of *Acetobacterium woodii* in AcetoScan mock-community analyses by Singh et al. (2020). Acetobacterium woodii is a known acetogen and, if it were misclassified as Romboutsia, the involvement of acetogens in VFA metabolism is further supported. Clostridium beijerinckii is a versatile acetogenic species since, in addition to acetate, it can produce ethanol, butyrate, etc. (Patakova et al., 2019).

Peptococcaceae bacterium was also observed (RA ~3–8%) while the VFA levels were high in reactors C1-DD1. This OTU, also found in C3-K1/2 and in the stable phase in C5-A1/3, were 84–92% similar to Peptococcaceae bacterium 1109, previously proposed to be a syntrophic organic acid-oxidising bacterium (Town et al., 2014; Town and Dumonceaux, 2016; Buettner et al., 2019; Wirth et al., 2019; Singh et al., 2021a). The OTU could also be related to the recently discovered and uncultured syntrophic propionate oxidising bacteria Candidatus Syntrophopropionicum ammoniitolerans isolated from propionate oxidising enrichments (Singh et al., 2021b). As this OTU was observed during the high levels of VFA (3–17 g/L) including propionate (2–10 g/L), its involvement in propionate degradation is further supported. Overall, the results obtained for C1-DD1 indicate a role of syntrophic organic-acid oxidising communities, which have been shown previously for reactors operating at high ammonia and VFA levels (Schnürer et al., 1994, 1996; Schnürer and Nordberg, 2008; Müller et al., 2016). During the VFA accumulation phase in C1-DD1, a decrease in RA of phylum Candidatus Clocimonetes was also observed. Disappearance of this phylum is suggested to be an indicator of disturbance (Calusinska et al., 2018; Klang et al., 2019; Poirier et al., 2020; Singh et al., 2021a), which is further supported by the results in this study. In the present analysis, it was observed that not all species belonging to this phylum were present in all types of reactors. Thus, the connection to process imbalance might need to be validated for each species within the phylum Candidatus Clocimonetes.

Reactor C2-VX1/2, which was operated with food waste and sewage sludge, showed somewhat different community structure from the other reactors. A community shift was also observed in this reactor around day 185, possibly due to a change in feed ratio of food waste and sewage sludge around that time. Among the dominant species, *Enteroscipio rubneri* was most abundant in C2-VX1/2, unlike in the other reactors. The OTU sequences represented by this species were observed to be ~80–86% similar to *Enteroscipio rubneri*. However, further analysis of the OTUs revealed that they were also associated with novel uncultured Clostridiaceae bacterium found by Campanaro et al. (2020), which is not yet validated. Since the reference database AcetoBase mostly contains sequences from validated bacterial species and AcetoScan identifies taxonomic relationships with the best-blast-hit strategy, the annotation for this species must be interpreted with caution.

The species *Acetitomaculum ruminis* was only observed (RA >3%) in the manure-supplemented reactors C3-K1/2 and C4-VS1/2. It is a known acetogen present in the rumen and performs reductive acetogenesis (Greening and Leedle, 1989; Le Van et al., 1998; Yang et al., 2015). An increase in RA of this species and decrease in RA of Cloacimonetes_HGW3 was observed prior to VFA accumulation (~day 175–217) in reactor C4-VS2, indicating a role in acetogenesis and syntrophic acid oxidation, respectively. *Lactobacillus antri*, observed in the same reactors, is a lactic acid-producing bacterium (Roos et al., 2005). Interestingly a decrease in its RA appeared to be related to an increase in RA of *Tepidanaerobacter syntrophicus*, followed by accumulation of VFA. *Tepidanaerobacter syntrophicus* is a syntrophic lactate/ethanol-degrading bacterium that produces acetate and is suggested to be an acetogen (Sekiguchi et al., 2006). These related events strongly indicate a role of the species in reductive acetogenesis in VFA metabolism in manure-based reactors.

Reactor C5-A1/3 was operated at 40–42°C with food waste and moderate to high ammonium-nitrogen conditions. Characteristic for this reactor was the presence of *Mahella australiensis*. This is a saccharolytic, (moderately) thermophilic bacterium producing not only acetate but also lactate, ethanol and H_2_/CO_2_ as end products (Bonilla Salinas et al., 2004). It is commonly reported in reactors operated with high protein substrates contents and an increase in ammonium-nitrogen might cause a decrease in relative abundance of this species (Niu et al., 2013; Zhang et al., 2021). Moreover, in this reactor the species *Thermoanaerobacter kivui* was detected. This is also a thermophilic acetogen (Leigh and Wolfe, 1983; Basen et al., 2018) generally not present in mesophilic reactors, but OTUs corresponding to this species (in C1-DD1 and C5-A1/3) were 80–93% similar to *Thermoanaerobacter kivui*. Potentially, this species could be the new uncultured bacteria also detected before in 16S rRNA gene amplicon analysis (Navarro et al., 2020) and recently identified in metagenomics analysis as a member of family Thermoanaerobacteraceae (Taxonomy ID: 2100788; Campanaro et al., 2020). The reactor operated with green waste (reactor C6-O1/2) specifically showed high RA (>3%) abundance of *Lagierella massiliensis* and *Varibaculum timonense* (renamed as *Urmitella timonensis*; GTDB, 2020), which were not detected in any other reactor. These bacteria belong to the poorly characterised order Tissierellales and might be involved in VFA metabolism, since other members of this order are known syntrophic organic acid-oxidising bacteria such as *Clostridium ultunense* (renamed as *Schnuerera ultunensis*; Schnürer et al., 1996; Oren and Garrity, 2020).

### Surveillance of Industrial Biogas Plants

In microbiological surveillance in this study, a disturbance-oriented long time series sampling strategy was adopted. Time series sampling was preferred over sampling with replicates because the sequential samples obtained in time series are as good as replicates in visualising changes in the community over time (Faust et al., 2015; Singh et al., 2021a). This was supported by the similar community composition and dynamics found in parallel reactors. Time series sampling also enabled simultaneous long-term surveillance of multiple reactors. The overall strategy adopted in the study helped overcome the low throughput barrier of FTHFS-based analytical methods (FTHFS gene T-RFLP, clone library or qPCR analysis), resolved the long duration dynamics of potential acetogenic and syntrophic communities and revealed changes in community structure before and during VFA accumulation. If community changes are closely followed in future studies, they can be used as an indicator of possible disturbance, which could help in the modification of operating parameters to avoid or minimise the impact of disturbance. Thus, the approach adequately meets the criteria for microbiological surveillance in biogas plants.

Further, the results from this study indicates that the applied method is able to target not only the potential acetogenic community but also syntrophic organic-acid oxidising bacterial communities *viz*. syntrophic acetate-oxidising bacteria (SAOB; phylum Cloacimonetes, *Tepidanaerobacter*, Peptococcaceae bacterium 1109; Sekiguchi et al., 2006; Westerholm et al., 2011b; Town et al., 2014; Town and Dumonceaux, 2016; Buettner et al., 2019; Wirth et al., 2019), syntrophic-propionate oxidising bacteria (phylum Cloacimonetes, family Peptococcaceae; Pelletier et al., 2008; Dyksma and Gallert, 2019; Singh et al., 2021b), syntrophic fatty-acid degraders (*Syntrophomonas*, *Syntrophothermus*; Sekiguchi et al., 2000; Zhang et al., 2004; Sousa et al., 2007) and syntrophic benzene degrading bacteria (family Peptococcaceae; van der Zaan et al., 2012; Gieg et al., 2014; Zhuang et al., 2015). Syntrophic organic-acid oxidising bacteria play a key role in the biogas process and even though not all being acetogens they can be detected due to the presence of FTHFS gene (Singh et al., 2019; Singh, 2021). The importance of both acetogenic and syntrophic bacterial communities in biogas process is well established and documented. Thus, combined visualization of these two communities makes our strategy a very strong tool for surveillance of biogas plants.

## Conclusion

Microbiological surveillance of biogas reactors was carried out by using FTHFS gene amplicon sequencing in an analysis of long time-series of weekly samples from commercial biogas plants operating with different substrates. The results obtained clearly visualised the diversity of the potential acetogenic community as well as several groups of syntrophic bacteria in biogas reactors based on different feed substrate/s and operating parameters and related to VFA metabolism. This targeted community may be involved in VFA metabolism, reductive acetogenesis and/or syntrophic acid oxidisation. The surveillance strategy also revealed changes in microbial communities before any significant changes were detectable in the physico-chemical profiles. These findings highlight the role of the targeted community in microbiological surveillance of biogas plants. Some potential indicators related to process disturbances were identified, findings which require validation. Overall, our FTHFS gene amplicon-based microbiological surveillance strategy demonstrated strong potential for use as a tool for monitoring the acetogenic and syntrophic community in biogas plants.

## Data Availability Statement

The datasets presented in this study can be found in online repositories. The names of the repository/repositories and accession number(s) can be found at: https://www.ncbi.nlm.nih.gov/, PRJNA723176.

## Author Contributions

ASc conceived the idea of the present study and was responsible for funding acquisition. ASi performed experiment and data analysis and wrote the manuscript with valuable help from all co-authors. JM and AB were responsible for collection of samples from the biogas plants and compiled corresponding metadata. All authors have read and agreed to the published version of the manuscript.

## Conflict of Interest

The authors declare that the research was conducted in the absence of any commercial or financial relationships that could be construed as a potential conflict of interest.

## Publisher’s Note

All claims expressed in this article are solely those of the authors and do not necessarily represent those of their affiliated organizations, or those of the publisher, the editors and the reviewers. Any product that may be evaluated in this article, or claim that may be made by its manufacturer, is not guaranteed or endorsed by the publisher.

## References

[ref1] Akuzawa M. Hori T. Haruta S. Ueno Y. Ishii M. Igarashi Y. (2011). Distinctive responses of metabolically active microbiota to acidification in a thermophilic anaerobic digester. Microb. Ecol. 61, 595–605. 10.1007/s00248-010-9788-1, PMID: 21240482

[ref2] Basen M. Geiger I. Henke L. Müller V. (2018). A genetic system for the thermophilic acetogenic bacterium thermoanaerobacter kivui. Appl. Environ. Microbiol. 84, e02210–e02217. 10.1128/AEM.02210-17, PMID: 29150512 PMC5772241

[ref3] Bonilla Salinas M. Fardeau M.-L. Thomas P. Cayol J.-L. Patel B. K. C. Ollivier B. (2004). Mahella australiensis gen. Nov., sp. nov., a moderately thermophilic anaerobic bacterium isolated from an Australian oil well. Int. J. Syst. Evol. Microbiol. 54, 2169–2173. 10.1099/ijs.0.02926-0, PMID: 15545453

[ref4] Borja R. Rincón B. (eds.) (2017). “Biogas production,” in Reference Module in Life Sciences (Elsevier), 785–798. 10.1016/B978-0-12-809633-8.09105-6

[ref5] Buettner C. von Bergen M. Jehmlich N. Noll M. (2019). Pseudomonas spp. are key players in agricultural biogas substrate degradation. Sci. Rep. 9:12871. 10.1038/s41598-019-49313-8, PMID: 31492882 PMC6731289

[ref6] Calusinska M. Goux X. Fossépré M. Muller E. E. L. Wilmes P. Delfosse P. (2018). A year of monitoring 20 mesophilic full-scale bioreactors reveals the existence of stable but different core microbiomes in bio-waste and wastewater anaerobic digestion systems. Biotechnol. Biofuels 11, 1–19. 10.1186/s13068-018-1195-8 30038663 PMC6052691

[ref7] Campanaro S. Treu L. Kougias P. G. De Francisci D. Valle G. Angelidaki I. (2016). Metagenomic analysis and functional characterization of the biogas microbiome using high throughput shotgun sequencing and a novel binning strategy. Biotechnol. Biofuels 9:26. 10.1186/s13068-016-0441-1, PMID: 26839589 PMC4736482

[ref8] Campanaro S. Treu L. Rodriguez-R L. M. Kovalovszki A. Ziels R. M. Maus I.. (2020). New insights from the biogas microbiome by comprehensive genome-resolved metagenomics of nearly 1600 species originating from multiple anaerobic digesters. Biotechnol. Biofuels 13:25. 10.1186/s13068-020-01679-y, PMID: 32123542 PMC7038595

[ref9] Collins M. D. Lawson P. A. Willems A. Cordoba J. J. Fernandez-Garayzabal J. Garcia P.. (1994). The phylogeny of the genus *Clostridium*: proposal of five new genera and eleven new species combinations. Int. J. Syst. Bacteriol. 44, 812–826. 10.1099/00207713-44-4-812, PMID: 7981107

[ref10] De Vrieze J. Saunders A. M. He Y. Fang J. Nielsen P. H. Verstraete W.. (2015). Ammonia and temperature determine potential clustering in the anaerobic digestion microbiome. Water Res. 75, 312–323. 10.1016/j.watres.2015.02.025, PMID: 25819618

[ref11] Drake H. L. Gößner A. S. Daniel S. L. (2008). Old acetogens, new light. Ann. N. Y. Acad. Sci. 1125, 100–128. 10.1196/annals.1419.016 18378590

[ref12] Drosg B. (2013). Process Monitoring in Biogas Plants. IEA Bioenergy. Available at: https://www.ieabioenergy.com/publications/process-monitoring-in-biogas-plants (Accessed November 16, 2020).

[ref13] Dyksma S. Gallert C. (2019). Candidatus Syntrophosphaera thermopropionivorans: a novel player in syntrophic propionate oxidation during anaerobic digestion. Environ. Microbiol. Rep. 11, 558–570. 10.1111/1758-2229.12759, PMID: 30985964

[ref14] Faust K. Lahti L. Gonze D. de Vos W. M. Raes J. (2015). Metagenomics meets time series analysis: unraveling microbial community dynamics. Curr. Opin. Microbiol. 25, 56–66. 10.1016/j.mib.2015.04.004 26005845

[ref15] Ferguson R. M. W. Coulon F. Villa R. (2018). Understanding microbial ecology can help improve biogas production in AD. Sci. Total Environ. 642, 754–763. 10.1016/j.scitotenv.2018.06.007 29920462

[ref16] Ferguson R. M. W. Villa R. Coulon F. (2014). Bioengineering options and strategies for the optimization of anaerobic digestion processes. Environ. Technol. Rev. 3, 1–14. 10.1080/09593330.2014.907362

[ref17] Fernández A. Huang S. Seston S. Xing J. Hickey R. Criddle C.. (1999). How stable is stable? Function versus community composition. Appl. Environ. Microbiol. 65, 3697–3704. 10.1128/AEM.65.8.3697-3704.1999, PMID: 10427068 PMC91553

[ref18] Gagen E. J. Denman S. E. Padmanabha J. Zadbuke S. Al Jassim R. Morrison M.. (2010). Functional gene analysis suggests different acetogen populations in the bovine rumen and tammar wallaby forestomach. Appl. Environ. Microbiol. 76, 7785–7795. 10.1128/AEM.01679-10, PMID: 20889794 PMC2988603

[ref19] Gagen E. J. Wang J. Padmanabha J. Liu J. de Carvalho I. P. C. Liu J.. (2014). Investigation of a new acetogen isolated from an enrichment of the tammar wallaby forestomach. BMC Microbiol. 14:314. 10.1186/s12866-014-0314-3, PMID: 25495654 PMC4275979

[ref20] Gieg L. M. Fowler S. J. Berdugo-Clavijo C. (2014). Syntrophic biodegradation of hydrocarbon contaminants. Curr. Opin. Biotechnol. 27, 21–29. 10.1016/j.copbio.2013.09.002, PMID: 24863893

[ref21] Greening R. C. Leedle J. A. Z. (1989). Enrichment and isolation of Acetitomaculum ruminis, gen. Nov., sp. nov.: acetogenic bacteria from the bovine rumen. Arch. Microbiol. 151, 399–406. 10.1007/BF00416597, PMID: 2500921

[ref22] GTDB (2020). Varibaculum timonense (Actinomycetaceae) reclassified to Urmitella timonensis (Tissierellaceae). GTDB. Available at: https://gtdb.ecogenomic.org/genomes?gid=GCF_900169515.1 (Accessed December 18, 2020).

[ref23] Güllert S. Fischer M. A. Turaev D. Noebauer B. Ilmberger N. Wemheuer B.. (2016). Deep metagenome and metatranscriptome analyses of microbial communities affiliated with an industrial biogas fermenter, a cow rumen, and elephant feces reveal major differences in carbohydrate hydrolysis strategies. Biotechnol. Biofuels 9, 1–20. 10.1186/s13068-016-0534-x 27279900 PMC4897800

[ref24] Hanreich A. Heyer R. Benndorf D. Rapp E. Pioch M. Reichl U.. (2012). Metaproteome analysis to determine the metabolically active part of a thermophilic microbial community producing biogas from agricultural biomass. Can. J. Microbiol. 58, 917–922. 10.1139/w2012-058, PMID: 22690648

[ref25] Henderson G. Leahy S. C. Janssen P. H. (2010). Presence of novel, potentially homoacetogenic bacteria in the rumen as determined by analysis of formyltetrahydrofolate synthetase sequences from ruminants. Appl. Environ. Microbiol. 76, 2058–2066. 10.1128/AEM.02580-09, PMID: 20118378 PMC2849231

[ref26] Heyer R. Benndorf D. Kohrs F. De Vrieze J. Boon N. Hoffmann M.. (2016). Proteotyping of biogas plant microbiomes separates biogas plants according to process temperature and reactor type. Biotechnol. Biofuels 9:155. 10.1186/s13068-016-0572-4, PMID: 27462366 PMC4960849

[ref27] Heyer R. Kohrs F. Benndorf D. Rapp E. Kausmann R. Heiermann M.. (2013). Metaproteome analysis of the microbial communities in agricultural biogas plants. New Biotechnol. 30, 614–622. 10.1016/j.nbt.2013.01.002, PMID: 23369865

[ref28] Hori T. Sasaki D. Haruta S. Shigematsu T. Ueno Y. Ishii M.. (2011). Detection of active, potentially acetate-oxidizing syntrophs in an anaerobic digester by flux measurement and formyltetrahydrofolate synthetase (FTHFS) expression profiling. Microbiology 157, 1980–1989. 10.1099/mic.0.049189-0, PMID: 21474532

[ref29] Klang J. Szewzyk U. Bock D. Theuerl S. (2019). Nexus between the microbial diversity level and the stress tolerance within the biogas process. Anaerobe 56, 8–16. 10.1016/j.anaerobe.2019.01.003, PMID: 30633970

[ref30] Kleinsteuber S. (2019). “Metagenomics of methanogenic communities in anaerobic digesters,” in Biogenesis of Hydrocarbons. eds. Stams A. J. M. Sousa D. Z. (Cham: Springer International Publishing), 337–359.

[ref31] Kohrs F. Heyer R. Magnussen A. Benndorf D. Muth T. Behne A.. (2014). Sample prefractionation with liquid isoelectric focusing enables in depth microbial metaproteome analysis of mesophilic and thermophilic biogas plants. Anaerobe 29, 59–67. 10.1016/j.anaerobe.2013.11.009, PMID: 24309213

[ref32] Kovács K. L. Kovács Á. T. Maróti G. Bagi Z. Csanádi G. Perei K.. (2004). Improvement of biohydrogen production and intensification of biogas formation. Rev. Environ. Sci. Biotechnol. 3, 321–330. 10.1007/s11157-004-7460-2

[ref33] Le Van T. D. Robinson J. A. Ralph J. Greening R. C. Smolenski W. J. Leedle J. A. Z.. (1998). Assessment of reductive acetogenesis with indigenous ruminal bacterium populations and Acetitomaculum ruminis. Appl. Environ. Microbiol. 64, 3429–3436. 10.1128/AEM.64.9.3429-3436.1998, PMID: 9726893 PMC106743

[ref34] Leaphart A. B. Lovell C. R. (2001). Recovery and analysis of formyltetrahydrofolate synthetase gene sequences from natural populations of acetogenic bacteria. Appl. Environ. Microbiol. 67, 1392–1395. 10.1128/AEM.67.3.1392-1395.2001, PMID: 11229939 PMC92742

[ref35] Leigh J. A. Wolfe R. S. (1983). Acetogenium kivui gen. Nov., sp. nov., a thermophilic acetogenic bacterium. Int. J. Syst. Bacteriol. 33:886. 10.1099/00207713-33-4-886

[ref36] Li Z. Henderson G. Yang Y. Li G. (2017). Diversity of formyltetrahydrofolate synthetase genes in the rumens of roe deer (*Capreolus pygargus*) and sika deer (*Cervus nippon*) fed different diets. Can. J. Microbiol. 63, 11–19. 10.1139/cjm-2016-0424, PMID: 27819479

[ref37] Luo G. Fotidis I. A. Angelidaki I. (2016). Comparative analysis of taxonomic, functional, and metabolic patterns of microbiomes from 14 full-scale biogas reactors by metagenomic sequencing and radioisotopic analysis. Biotechnol. Biofuels 9:51. 10.1186/s13068-016-0465-6, PMID: 26941838 PMC4776419

[ref38] Madsen M. Holm-Nielsen J. B. Esbensen K. H. (2011). Monitoring of anaerobic digestion processes: a review perspective. Renew. Sust. Energ. Rev. 15, 3141–3155. 10.1016/j.rser.2011.04.026

[ref39] Maus I. Koeck D. E. Cibis K. G. Hahnke S. Kim Y. S. Langer T.. (2016). Unraveling the microbiome of a thermophilic biogas plant by metagenome and metatranscriptome analysis complemented by characterization of bacterial and archaeal isolates. Biotechnol. Biofuels 9:171. 10.1186/s13068-016-0581-3, PMID: 27525040 PMC4982221

[ref40] McMurdie P. J. Holmes S. (2013). Phyloseq: an R package for reproducible interactive analysis and graphics of microbiome census data. PLoS One 8:e61217. 10.1371/journal.pone.0061217, PMID: 23630581 PMC3632530

[ref41] Moestedt J. Müller B. Westerholm M. Schnürer A. (2016). Ammonia threshold for inhibition of anaerobic digestion of thin stillage and the importance of organic loading rate. Microb. Biotechnol. 9, 180–194. 10.1111/1751-7915.12330, PMID: 26686366 PMC4767286

[ref42] Müller B. Sun L. Westerholm M. Schnürer A. (2016). Bacterial community composition and fhs profiles of low- and high-ammonia biogas digesters reveal novel syntrophic acetate-oxidising bacteria. Biotechnol. Biofuels 9, 1–18. 10.1186/s13068-016-0454-9 26925165 PMC4769498

[ref43] Navarro R. R. Otsuka Y. Matsuo K. Sasaki K. Sasaki K. Hori T.. (2020). Combined simultaneous enzymatic saccharification and comminution (SESC) and anaerobic digestion for sustainable biomethane generation from wood lignocellulose and the biochemical characterization of residual sludge solid. Bioresour. Technol. 300:122622. 10.1016/j.biortech.2019.122622, PMID: 31891856

[ref44] Nierychlo M. Andersen K. S. Xu Y. Green N. Jiang C. Albertsen M.. (2020). MiDAS 3: an ecosystem-specific reference database, taxonomy and knowledge platform for activated sludge and anaerobic digesters reveals species-level microbiome composition of activated sludge. Water Res. 182:115955. 10.1016/j.watres.2020.115955, PMID: 32777640

[ref45] Niu Q. Qiao W. Qiang H. Li Y.-Y. (2013). Microbial community shifts and biogas conversion computation during steady, inhibited and recovered stages of thermophilic methane fermentation on chicken manure with a wide variation of ammonia. Bioresour. Technol. 146, 223–233. 10.1016/j.biortech.2013.07.038, PMID: 23934339

[ref46] Ohashi Y. Igarashi T. Kumazawa F. Fujisawa T. (2007). Analysis of acetogenic bacteria in human feces with formyltetrahydrofolate synthetase sequences. Biosci. Microflora 26, 37–40. 10.12938/bifidus.26.37

[ref47] Oksanen J. Blanchet F. G. Friendly M. Kindt R. Legendre P. McGlinn D.. (2019). *Vegan*: Community Ecology Package. Version 2.5–6; Comprehensive R Archive Network (CRAN). Available at: https://cran.r-project.org/package=vegan

[ref48] Oren A. Garrity G. (2020). List of new names and new combinations previously effectively, but not validly, published. Int. J. Syst. Evol. Microbiol. 70, 4043–4049. 10.1099/ijsem.0.004244, PMID: 32731908

[ref49] Ortseifen V. Stolze Y. Maus I. Sczyrba A. Bremges A. Albaum S. P.. (2016). An integrated metagenome and -proteome analysis of the microbial community residing in a biogas production plant. J. Biotechnol. 231, 268–279. 10.1016/j.jbiotec.2016.06.014 27312700

[ref50] Patakova P. Branska B. Sedlar K. Vasylkivska M. Jureckova K. Kolek J.. (2019). Acidogenesis, solventogenesis, metabolic stress response and life cycle changes in Clostridium beijerinckii NRRL B-598 at the transcriptomic level. Sci. Rep. 9:1371. 10.1038/s41598-018-37679-0, PMID: 30718562 PMC6362236

[ref51] Pelletier E. Kreimeyer A. Bocs S. Rouy Z. Gyapay G. Chouari R.. (2008). “*Candidatus* Cloacamonas acidaminovorans”: genome sequence reconstruction provides a first glimpse of a new bacterial division. J. Bacteriol. 190, 2572–2579. 10.1128/JB.01248-07 18245282 PMC2293186

[ref52] Pester M. Brune A. (2006). Expression profiles of *fhs* (FTHFS) genes support the hypothesis that spirochaetes dominate reductive acetogenesis in the hindgut of lower termites. Environ. Microbiol. 8, 1261–1270. 10.1111/j.1462-2920.2006.01020.x, PMID: 16817934

[ref53] Poirier S. Déjean S. Midoux C. Lê Cao K.-A. Chapleur O. (2020). Integrating independent microbial studies to build predictive models of anaerobic digestion inhibition by ammonia and phenol. Bioresour. Technol. 316:123952. 10.1016/j.biortech.2020.123952, PMID: 32771938

[ref54] R Core Team (2013). R: A Language and Environment for Statistical Computing. R Foundation for Statistical Computing; Vienna, Austria. Available at: http://www.r-project.org

[ref55] Robles G. Nair R. B. Kleinsteuber S. Nikolausz M. Sárvári Horváth I. (2018). “Biogas production: microbiological aspects,” in Biogas: Fundamentals, Process, and Operation. eds. Tabatabaei M. Ghanavati H. (Cham: Springer International Publishing), 163–198.

[ref56] Roos S. Engstrand L. Jonsson H. (2005). Lactobacillus gastricus sp. nov., lactobacillus antri sp. nov., lactobacillus kalixensis sp. nov. and lactobacillus ultunensis sp. nov., isolated from human stomach mucosa. Int. J. Syst. Evol. Microbiol. 55, 77–82. 10.1099/ijs.0.63083-0, PMID: 15653856

[ref57] RStudio Team (2020). RStudio: Integrated Development Environment for R. Boston, MA: RStudio, PBC. Available at: http://www.rstudio.com/

[ref58] Saheb-Alam S. Singh A. Hermansson M. Persson F. Schnürer A. Wilén B.-M.. (2017). Effect of start-up strategies and electrode materials on carbon dioxide reduction on biocathodes. Appl. Environ. Microbiol. 84, e02242–e02317. 10.1128/AEM.02242-17, PMID: 29222104 PMC5795077

[ref59] Schlüter A. Bekel T. Diaz N. N. Dondrup M. Eichenlaub R. Gartemann K.-H.. (2008). The metagenome of a biogas-producing microbial community of a production-scale biogas plant fermenter analysed by the 454-pyrosequencing technology. J. Biotechnol. 136, 77–90. 10.1016/j.jbiotec.2008.05.008, PMID: 18597880

[ref60] Schnürer A. (2016). “Biogas production: microbiology and technology,” in Advances in Biochemical Engineering/Biotechnology. eds. Hatti-Kaul R. Mamo G. Mattiasson B. (Cham: Springer International Publishing), 195–234. 10.1007/10_2016_5 27432246

[ref61] Schnürer A. Houwen F. P. Svensson B. H. (1994). Mesophilic syntrophic acetate oxidation during methane formation by a triculture at high ammonium concentration. Arch. Microbiol. 162, 70–74. 10.1007/BF00264375

[ref62] Schnürer A. Jarvis Å. (2017). Microbiology of the Biogas Process. Swedish University of Agricultural Sciences. Available at: https://www.researchgate.net/publication/327388476_Microbiology_of_the_biogas_process

[ref63] Schnürer A. Nordberg Å. (2008). Ammonia, a selective agent for methane production by syntrophic acetate oxidation at mesophilic temperature. Water Sci. Technol. 57, 735–740. 10.2166/wst.2008.097, PMID: 18401146

[ref64] Schnürer A. Schink B. Svensson B. H. (1996). *Clostridium ultunense* sp. nov., a mesophilic bacterium oxidizing acetate in syntrophic association with a hydrogenotrophic methanogenic bacterium. Int. J. Syst. Bacteriol. 46, 1145–1152. 10.1099/00207713-46-4-1145, PMID: 8863449

[ref65] Sekiguchi Y. Imachi H. Susilorukmi A. Muramatsu M. Ohashi A. Harada H.. (2006). Tepidanaerobacter syntrophicus gen. Nov., sp. nov., an anaerobic, moderately thermophilic, syntrophic alcohol- and lactate-degrading bacterium isolated from thermophilic digested sludges. Int. J. Syst. Evol. Microbiol. 56, 1621–1629. 10.1099/ijs.0.64112-0, PMID: 16825639

[ref66] Sekiguchi Y. Kamagata Y. Nakamura K. Ohashi A. Harada H. (2000). Syntrophothermus lipocalidus gen. Nov., sp. nov., a novel thermophilic, syntrophic, fatty-acid-oxidizing anaerobe which utilizes isobutyrate. Int. J. Syst. Evol. Microbiol. 50, 771–779. 10.1099/00207713-50-2-771, PMID: 10758888

[ref67] Singh A. (2020). Genomic DNA Extraction From Anaerobic Digester Samples. protocols.io. 10.17504/protocols.io.bgxkjxkw.

[ref68] Singh A. (2021). Microbiological Surveillance of Biogas Plants: Focusing on the Acetogenic Community. Available at: https://pub.epsilon.slu.se/22699/10.3389/fmicb.2021.700256 PMC8415747 34484143

[ref69] Singh A. Müller B. Fuxelius H.-H. Schnürer A. (2019). AcetoBase: a functional gene repository and database for formyltetrahydrofolate synthetase sequences. Database 2019:baz142. 10.1093/database/baz142, PMID: 31832668 PMC6908459

[ref70] Singh A. Müller B. Schnürer A. (2021a). Profiling temporal dynamics of acetogenic communities in anaerobic digesters using next-generation sequencing and T-RFLP. Sci. Rep. 11:13298. 10.1038/s41598-021-92658-2 34168213 PMC8225771

[ref71] Singh A. Nylander J. A. A. Schnürer A. Bongcam-Rudloff E. Müller B. (2020). High-throughput sequencing and unsupervised analysis of formyltetrahydrofolate synthetase (FTHFS) gene amplicons to estimate acetogenic community structure. Front. Microbiol. 11:2066. 10.3389/fmicb.2020.02066, PMID: 32983047 PMC7481360

[ref72] Singh A. Schnürer A. Westerholm M. (2021b). Enrichment and description of novel bacteria performing syntrophic propionate oxidation at high ammonia level. Environ. Microbiol. 23, 1620–1637. 10.1111/1462-2920.15388 33400377

[ref73] Sousa D. Z. Smidt H. Madalena Alves M. Stams A. J. M. (2007). Syntrophomonas zehnderi sp. nov., an anaerobe that degrades long-chain fatty acids in co-culture with Methanobacterium formicicum. Int. J. Syst. Evol. Microbiol. 57, 609–615. 10.1099/ijs.0.64734-0, PMID: 17329794

[ref74] Theuerl S. Klang J. Prochnow A. (2019). Process disturbances in agricultural biogas production—causes, mechanisms and effects on the biogas microbiome: a review. Energies 12:365. 10.3390/en12030365

[ref75] Town J. R. Dumonceaux T. J. (2016). Laboratory-scale bioaugmentation relieves acetate accumulation and stimulates methane production in stalled anaerobic digesters. Appl. Microbiol. Biotechnol. 100, 1009–1017. 10.1007/s00253-015-7058-3, PMID: 26481626 PMC4703610

[ref76] Town J. R. Links M. G. Fonstad T. A. Dumonceaux T. J. (2014). Molecular characterization of anaerobic digester microbial communities identifies microorganisms that correlate to reactor performance. Bioresour. Technol. 151, 249–257. 10.1016/j.biortech.2013.10.070, PMID: 24246480

[ref77] Treu L. Kougias P. G. Campanaro S. Bassani I. Angelidaki I. (2016). Deeper insight into the structure of the anaerobic digestion microbial community; the biogas microbiome database is expanded with 157 new genomes. Bioresour. Technol. 216, 260–266. 10.1016/j.biortech.2016.05.081, PMID: 27243603

[ref78] UGC (2018). Next Generation Sequencing at Uppsala Genome Center (UGC). Uppsala: Uppsala Genome Center, Science for Life Lab; Sweden. Available at: https://www.scilifelab.se

[ref79] van der Zaan B. M. Saia F. T. Stams A. J. M. Plugge C. M. de Vos W. M. Smidt H.. (2012). Anaerobic benzene degradation under denitrifying conditions: peptococcaceae as dominant benzene degraders and evidence for a syntrophic process. Environ. Microbiol. 14, 1171–1181. 10.1111/j.1462-2920.2012.02697.x, PMID: 22296107

[ref80] Ward A. J. Hobbs P. J. Holliman P. J. Jones D. L. (2008). Optimisation of the anaerobic digestion of agricultural resources. Bioresour. Technol. 99, 7928–7940. 10.1016/j.biortech.2008.02.044, PMID: 18406612

[ref81] Westerholm M. Müller B. Arthurson V. Schnürer A. (2011a). Changes in the acetogenic population in a mesophilic anaerobic digester in response to increasing ammonia concentration. Microbes Environ. 26, 347–353. 10.1264/jsme2.ME11123 21869569

[ref82] Westerholm M. Müller B. Singh A. Karlsson Lindsjö O. Schnürer A. (2018). Detection of novel syntrophic acetate-oxidizing bacteria from biogas processes by continuous acetate enrichment approaches. Microb. Biotechnol. 11, 680–693. 10.1111/1751-7915.13035, PMID: 29239113 PMC6011928

[ref83] Westerholm M. Roos S. Schnürer A. (2011b). *Tepidanaerobacter acetatoxydans* sp. nov., an anaerobic, syntrophic acetate-oxidizing bacterium isolated from two ammonium-enriched mesophilic methanogenic processes. Syst. Appl. Microbiol. 34, 260–266. 10.1016/j.syapm.2010.11.018 21498020

[ref84] Wirth R. Böjti T. Lakatos G. Maróti G. Bagi Z. Rákhely G.. (2019). Characterization of core microbiomes and functional profiles of mesophilic anaerobic digesters fed with *Chlorella vulgaris* green microalgae and maize silage. Front. Energy Res. 7:111. 10.3389/fenrg.2019.00111

[ref85] Wolf C. McLoone S. Bongards M. (2009). Biogas plant control and optimization using computational intelligence methods (biogasanlagenregelung und -optimierung mit computational intelligence methoden). Automatisierungstechnik 57, 638–649. 10.1524/auto.2009.0809

[ref86] Yang C.-L. Guan L.-L. Liu J.-X. Wang J.-K. (2015). Rumen fermentation and acetogen population changes in response to an exogenous acetogen TWA4 strain and *Saccharomyces cerevisiae* fermentation product. J Zhejiang Univ Sci B 16, 709–719. 10.1631/jzus.B1500013, PMID: 26238546 PMC4534548

[ref87] Yoshida K. Shimizu N. (2020). Biogas production management systems with model predictive control of anaerobic digestion processes. Bioprocess Biosyst. Eng. 43, 2189–2200. 10.1007/s00449-020-02404-7, PMID: 32683505 PMC7581619

[ref88] Zhang C. Liu X. Dong X. (2004). Syntrophomonas curvata sp. nov., an anaerobe that degrades fatty acids in co-culture with methanogens. Int. J. Syst. Evol. Microbiol. 54, 969–973. 10.1099/ijs.0.02903-0, PMID: 15143051

[ref89] Zhang L. Mou A. Guo B. Sun H. Anwar M. N. Liu Y. (2021). Simultaneous phosphorus recovery in energy generation reactor (SPRING): high rate thermophilic Blackwater treatment. Resour. Conserv. Recycl. 164:10516. 10.1016/j.resconrec.2020.105163

[ref90] Zhuang L. Tang J. Wang Y. Hu M. Zhou S. (2015). Conductive iron oxide minerals accelerate syntrophic cooperation in methanogenic benzoate degradation. J. Hazard. Mater. 293, 37–45. 10.1016/j.jhazmat.2015.03.039, PMID: 25827267

